# Tim-3 Expression and MGMT Methylation Status Association With Survival in Glioblastoma

**DOI:** 10.3389/fphar.2020.584652

**Published:** 2020-09-15

**Authors:** Ji Zhang, Ke Sai, Xiao li Wang, Sheng quan Ye, Li jiao Liang, Yi Zhou, Zhi jie Chen, Wan-Ming Hu, Jian min Liu

**Affiliations:** ^1^ Department of Neurosurgery, State Key Laboratory of Oncology in South China, Collaborative Innovation Center for Cancer Medicine, Sun Yat-sen University Cancer Center, Guangzhou, China; ^2^ Department of General Surgery, Shang Jin Nan Fu Hospital of West China Hospital of Sichuan University, Chengdu, China; ^3^ Department of Anesthesiology, Sun Yat-sen University Cancer Center, State Key Laboratory of Oncology in South China, Collaborative Innovation Center for Cancer Medicine, Guangzhou, China; ^4^ Department of Pathology, Sun Yat-sen University Cancer Center, State Key Laboratory of Oncology in South China, Collaborative Innovation Center for Cancer Medicine, Guangzhou, China; ^5^ Department of Neurosurgery, The First Affiliated Hospital of Guangzhou University of Traditional Chinese Medicine, Guangzhou, China

**Keywords:** glioblastoma multiforme, O-6-methylguanine-DNA methyltransferase, prognosis, immune, T cell immunoglobulin mucin-3

## Abstract

**Background:**

A profound understanding of the molecular landscape of glioblastoma multiforme (GBM) will make it possible to develop better and more intelligent therapies directed toward specific molecular targets and may one day yield better prognostic capabilities. Immune checkpoint molecules have inspired the emergence of immune checkpoint-targeting therapeutic strategies. However, the prognostic significance of the immune checkpoint molecule T cell immunoglobulin mucin-3 (Tim-3) on tumor-infiltrating immune cells (TIICs) and O-6-methylguanine-DNA methyltransferase (MGMT) promoter methylation status has not yet been fully elucidated. We aimed to develop an MGMT promoter methylation status-associated immune prognostic signature for GBM.

**Patients and Methods:**

A total of 84 patients with newly diagnosed GBM were included in this study. MGMT promoter methylation status was retrospectively analyzed, and the expression level of Tim-3 was investigated using immunohistochemistry (IHC). The correlation between Tim-3 expression combined with MGMT promoter methylation status and prognosis was explored.

**Results:**

Tim-3 expression varied in GBM patients. Mesenchymal expression of Tim-3 in GBM tissues was present 73.81% (62/84) of patients, and these were subdivided into groups based on low 15.48% (13/84), moderate 7.14% (6/84), or strong expression 51.19% (43/84). Forty-eight patients had tumors that tested positive for MGMT promoter methylation, while the remaining 36 patients tested negative.

**Conclusions:**

We profiled the immune status of MGMT promoter methylation in GBM and established a local immune signature for GBM that could independently identify patients with a favorable prognosis, indicating a relationship between prognosis and GBM immune signature. MGMT promoter methylation with lower Tim-3 expression was significantly associated with better survival.

## Introduction

Glioblastoma is the most common and devastating primary brain tumor in adults (Wirsching et al., 2016). Despite recent advances, only a few treatment strategies are available for GBMs, and their outcomes remain dismal (Stupp et al., 2015). There are few effective treatment options for GBMs, and these carry high risks of relapse and short survival periods. Because the biology of GBM at the cellular and molecular levels is not well understood, especially in relation to treatment, the development of novel therapeutic approaches requires a deeper understanding of the tumor’s nature (Yu et al., 2017). In addition to standard treatment involving surgery, radiotherapy and chemotherapy, immunotherapy has been rapidly identified as a promising modality to treat GBM (Deng et al., 2019). A number of immune-related parameters have been reported to be predictive of outcomes for patients with GBM (Han et al., 2014; Han et al., 2015). In particular, MGMT promoter methylation status was reported to be significantly related to GBM prognosis (Rao et al., 2018). However, there is still a lack of studies that systematically explore the effects of MGMT promoter methylation status on the immune microenvironment and on the associations between MGMT promoter methylations status, immune microenvironment, and prognosis.

Tim-3 is widely expressed by mature T lymphocytes and macrophages (Sabatos et al., 2003). Of note, with the exception of the immune response, increasing evidence has suggested that Tim-3 has functional roles in tumor biology (Yan et al., 2015). Previous studies suggest that Tim-3 is a negative immune regulator that may be upregulated in the GBM tumor environment, so Tim-3 is a promising target in glioma treatment. However, until now, no evidence has revealed the value of Tim-3 as a prognostic biomarker in GBM patients. The present study aimed to investigate the influence of MGMT promoter methylation on the immune microenvironment and to develop an MGMT-associated immune prognostic signature for GBM.

## Materials and Methods

### Patients and Specimens

A cohort of patients with newly histologically diagnosed GBM (WHO grade IV) was studied consecutively from July 2016 to January 2018. We only included patients for whom affirmatory MGMT promoter methylation status, treatment course, and survival outcome were known. Patients with a mixed history of cancer other than GBM and previous adjuvant radiotherapy or chemotherapy were excluded. Patients who died of diseases unrelated to glioma were also excluded from the study. Patient age ranged from 18 to 70 years at the time of diagnosis. Neurological status was assessed before and after neurosurgery, and Karnofsky performance status (KPS) was not less than 70 in all patients. A series of 84 eligible patients who had tumor tissue available for testing were included in this study. These patients received standard subsequent treatment according to the Stupp protocol (Stupp et al., 2005). Follow-up was carried out regularly. The overall survival (OS) was defined as the interval from GBM diagnosis until either death or, for those who were removed, until the last known follow-up.

### Immunohistochemistry (IHC)

Tim-3 was immunohistochemically stained using a previously described standard technique (Li et al., 2018). Briefly, slides were deparaffinized in xylene and rehydrated in graded alcohol. Antigen retrieval was performed in tris-ethylenediaminetetraacetic acid (EDTA; pH 9.0) buffer at 95°C for 20 min. Slides were incubated in tris-buffered saline (TBS) for 5 min. Endogenous peroxidase blocking was performed in 3% H_2_O_2_ for 10 min. Subsequently, the slides were incubated in a rabbit polyclonal antibody against Tim-3 (1:500; Abcam, Inc., Cambridge, MA) overnight at 4°C. The slides were rinsed five times with 0.01 M phosphate-buffered saline (PBS; pH 7.4) for 10 min. Sections were incubated with primary antibodies against Tim-3 (1:1,000; catalog no. ab185703, Abcam) and with a horseradish peroxidase (HRP)–tagged secondary antibody (1:1,000; catalog no. sc-3836, Santa Cruz Biotechnology, Inc.) for another 1 hour at 37°C. Subsequently, the slides were washed in PBS and stained with 3,3-diaminobenzidine (DAB). Finally, the slides were counterstained, dehydrated, and mounted.

### IHC Assessment

The degree of Tim-3 protein expression was independently reviewed by two neuropathologists. The number of stained cells was designated as unexpressed (0), weak (1–5 cells/HPF), moderate (5–10 cells/HPF), or strong (>10 cells/HPF). The model of the microscope was a BX53, Olympus.

### MGMT Promoter Methylation

For the subset of patients who had documented positive MGMT promoter methylation of their initially resected tumor tissues, MGMT promoter methylation was further confirmed by methylation-specific real-time PCR, according to our institutional practice.

### Statistical Analysis

Statistical analyses were performed using GraphPad Prism version 7.0.0. The correlation between Tim-3 expression intensity combined with MGMT promoter methylation status and prognosis was calculated using a chi-square test. Spearman’s correlation analysis and the corresponding statistical significance were used to evaluate correlations with gene expression. Independent prognostic factors for OS were identified using the Cox’s proportional hazards model. The OS curves were plotted using the Kaplan-Meier method, and log-rank tests were employed to assess the resulting survival curves. A probability value of less than 0.05 was regarded as significant.

## Results

### Clinicopathological Characteristics

Archival tissue samples from 84 patients with GBM were enrolled in the study. Of the 84 GBM patients, 43 were men (51.19%) and 41 were women (49.81%). The median age at diagnosis was 41 years (range, 18–70 years), and the median KPS was 90 (range, 70–100). All specimens were obtained from the supratentorial area as identified by preoperative MRI. The follow-up duration ranged from 4 to 47 months, and the median OS was 17.3 months.

### Tim-3 Expression

**Figure 1:**
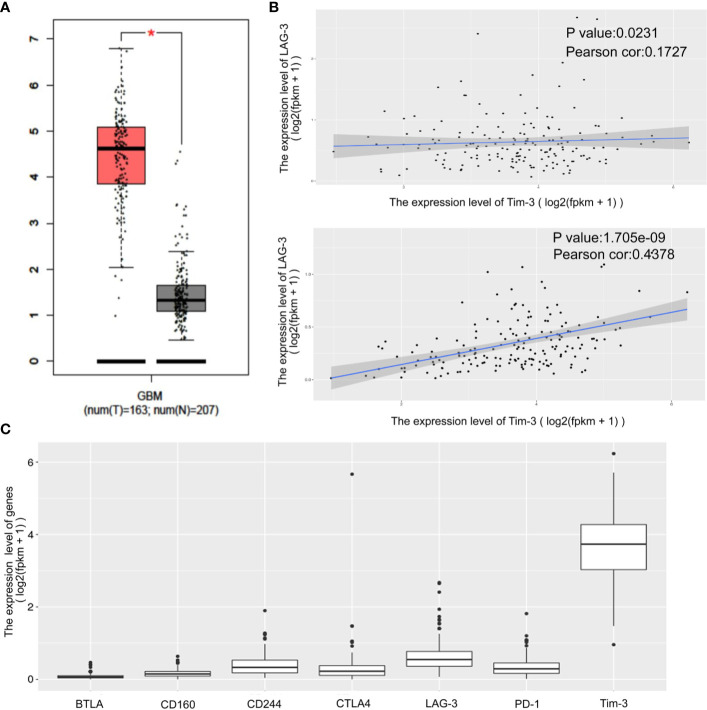
Differential expression of the immune checkpoint molecules PD-1, CTLA4, Tim-3 and LAG-3 in GBM tissues compared to corresponding normal adjacent tissues by TIMER analysis. Tim-3 expression in GBM compare with normal adjacent tissues **(A)**. Tim-3 expression is significantly associated with LAG-3 and PD-1 in GBM according to the TCGA database **(B)**. Tim-3 is one of the genes expressed differentially in exhausted T cells in GBM according to the TCGA database **(C)**.

**Figure 2:**
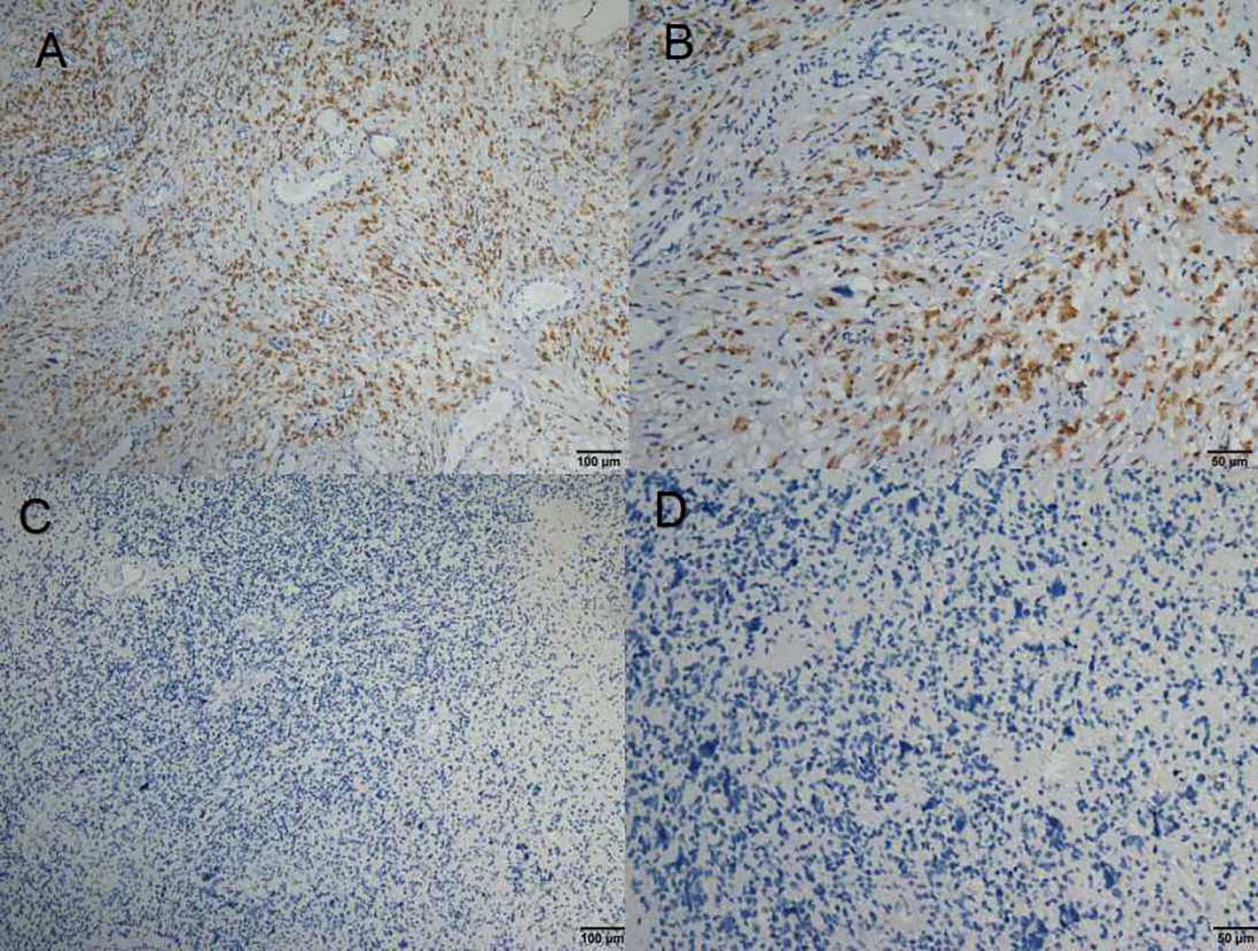
**(A–D)** Immunohistochemical staining of Tim-3 expression in formalin-fixed, paraffin-embedded GBM tissues.

The expression status of specific inhibitory receptors, including Tim-3, programmed cell death 1 (PD-1), cytotoxic T-lymphocyte-associated protein 4 (CTLA4), and lymphocyte-activation gene 3 (LAG-3), are associated with T cell exhaustion and immune escape. The Oncomine database was used to investigate the mRNA levels of these molecules in GBMs and normal tissues (**Figure 1C**). As shown in **Figure 1A**, Tim-3 expression was markedly higher in GBM than in corresponding normal tissue. To validate the relationships between the immune checkpoint molecules Tim-3 and LAG-3 and between Tim-3 and PD-1, RNA-seq data from the TCGA database (https://cancergenome.nih.gov/) were analyzed. As shown in **Figure 1B**, with a p-value threshold of 0.05, Tim-3 expression was significantly related to both LAG-3 and PD-1 expression in GBM. Further, Tim-3 and other exhausting immune molecules (LAG-3, PD-1, CTLA4, CD244, and PD-1) exhibited significantly different expression profiles between normal brain tissues and GBM tissues according to the TCGA database, indicating their potential correlation with glioma progression (**Figure 1**). According to our immunohistochemical analysis, mesenchymal expression of the immune checkpoint molecule Tim-3 in tumor-infiltrating immune cells (TIICs) was not observed in 22/84 patients (26.19%), weak in 13/84 patients (15.48%), moderate in 6/84 patients (7.14%), and strong in 43/84 patients (51.19%) (**Figure 2** and **Table 1**). Notably, strong expression of Tim-3 was more frequently observed in GBM than it was in other tissues.

**Table 1:** Tim-3 expression in 84 GBM samples.

No. of cases (84)	NE (22)	Tim-3 expression		Strong (43)
		Weak (13)	Moderate (6)	
Methylation	14	10	2	22
Non-methylation	8	3	4	21

NE, no expression.

### MGMT Methylation Status in 84 GBM Samples

**Figure 3:**
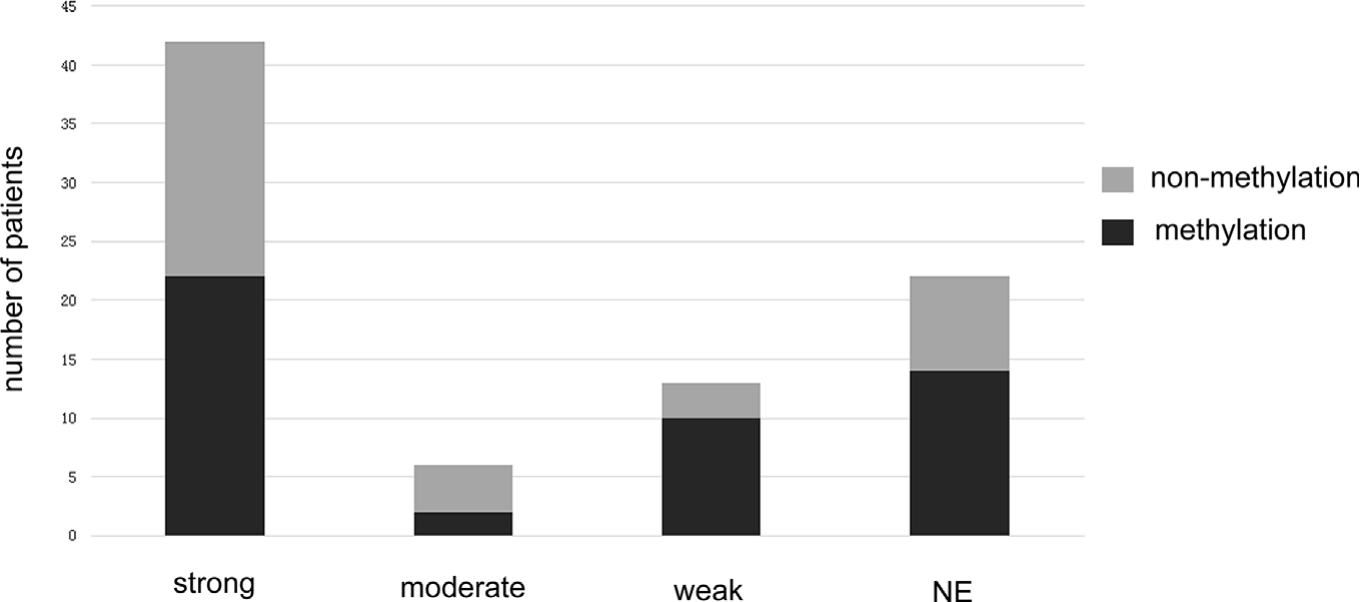
The bar graph shows the methylated and unmethylated MGMT distributions in different Tim-3 expression groups.

MGMT methylation status was analyzed for the 84 patients included in this study. All patients had MGMT promoter methylation test results. Testing was performed *via* methylation specific real-time PCR at the time of diagnosis. Forty-eight patients (57.14%) were determined to have MGMT methylated tumors, and 36 patients (42.76%) were determined to have MGMT unmethylated tumors (**Figure 3** and **Table 1**).

### Correlations Between MGMT Methylation Status Combined With Tim-3 Expression and Survival

**Figure 4:**
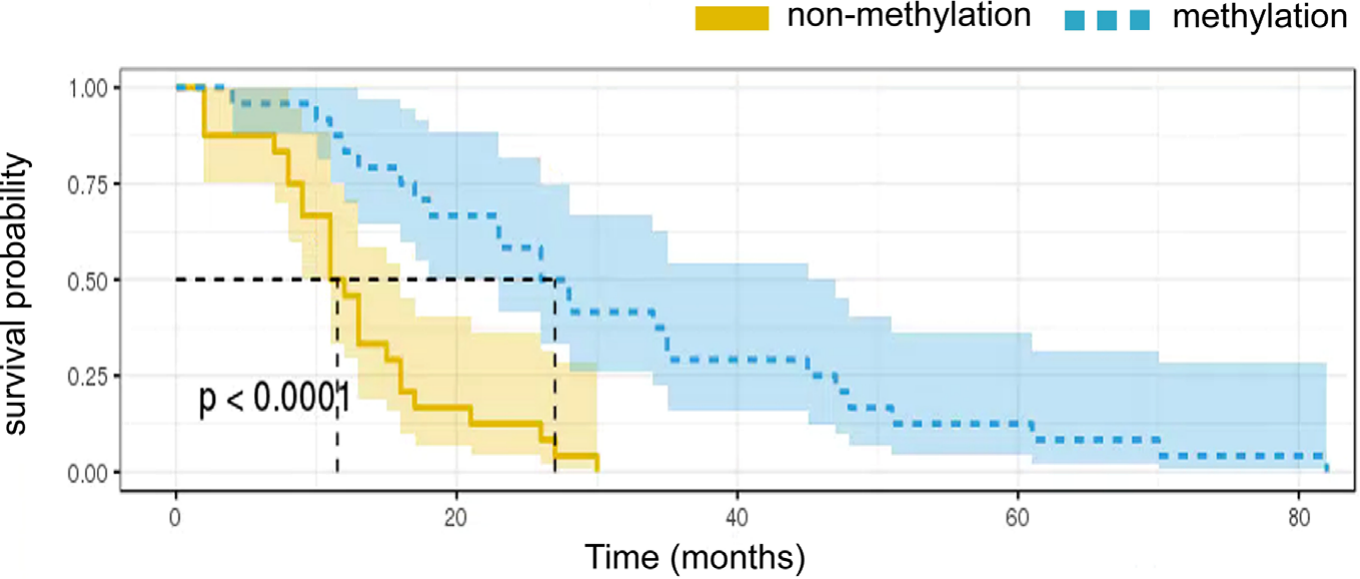
Kaplan-Meier survival curves showing overall survival according to MGMT promoter methylation status.

The relationship between Tim-3 expression and MGMT promoter methylation status is shown in **Table 1** and **Figure 3**. A low expression of Tim-3 in TIICs was associated with MGMT promoter methylation status. Univariate analysis revealed a significant correlation between low expression of Tim-3 in TIICs in combination with MGMT promoter methylation and a better prognosis. We summarized the correlation between Tim-3 expression combined with MGMT promoter methylation and GBM patient prognosis (**Figure 4**). Increased expression of Tim-3 combined with MGMT promoter nonmethylation was significantly associated with a poor prognosis. No significant correlations between Tim-3 expression level and either gender (P = 0.846) or tumor location (P = 0.447) were observed. However, moderate or strong expression of Tim-3 with either MGMT promoter methylation or nonmethylation was associated with a poor prognosis. In patients with no Tim-3 expression in combination with MGMT promoter nonmethylation, there was a similar association with prognosis, while MGMT promoter methylation was associated with a good prognosis.

Multivariate Cox regression analyses confirmed that a combination of Tim-3 expression and MGMT promoter methylation status was an independent risk factor for survival in GBM patients. Strong expression of Tim-3 in combination with MGMT promoter nonmethylation correlated significantly with shorter OS in each of the four subgroups (p < 0.05, **Figure 4**). The median survival was 16.9 and 16.4 months for patients whose tumor had unexpressed and moderate levels of Tim-3, respectively, whereas the median survival was 7.6 months for those who showed high levels of Tim-3 expression and MGMT promoter nonmethylation. In patients with low expression of Tim-3 and with MGMT promoter methylation, the average survival time was 21.8 months.

## Discussion

Glioblastoma is the most common and lethal primary brain tumor, with a high risk of recurrence and a short survival period, and finding the cure for this formidable disease is a daunting task (Stupp et al., 2015; Li et al., 2018). Recent developments in glioblastoma research emphasize targeting the molecular characteristics of the tumor as well as various approaches related to immunotherapy. Many new molecular markers have been identified, but MGMT promoter methylation status in particular is commonly used in GBM studies (Guo et al., 2019). 

The discovery that differential MGMT promoter methylation in GBM plays a key role in the understanding of glioma biology (Chai et al., 2019). Increasing numbers of studies indicate that the tumor immunological microenvironments of gliomas differ based on their molecular properties (Berghoff et al., 2017). However, the mechanism that regulates the relationship between MGMT promoter methylation status and the immune microenvironment is still unknown. The current study systematically investigated the prognostic impact of GBM of immune checkpoint molecule Tim-3 expression and MGMT promoter methylation status in TIICs. We identified Tim-3 expression in combination with MGMT promoter methylation status as a novel prognostic parameter for GBM. MGMT promoter methylation status was related to Tim-3 expression in immune cell infiltrating GBM. Our data demonstrated that Tim-3 is differentially expressed in most GBM tissues. Further, we observed that the checkpoint molecule Tim-3 in combination with MGMT promoter methylation status showed significant prognostic potential. Interestingly, strong expression of Tim-3 in combination with MGMT promoter nonmethylation showed a poor effect on survival. Thus, expression of Tim-3 with MGMT promoter methylation status has potential to be a prognostic predictor in immune cell infiltrating GBM.

To the best of our knowledge, the present study is the first to report that Tim-3 expression in combination with MGMT promoter methylation status is a critical prognostic variable for patients with GBM. Tim-3 is an immune regulatory molecule that motivates downstream cascade events upon stimulation by its ligand (Liu et al., 2014; Yan et al., 2015). Emerging research has demonstrated the importance of Tim-3 in human tumorigenesis. However, no studies have been performed which investigate the role of Tim-3 expression in combination with MGMT promoter methylation status in GBM patient prognosis. Aberrant expression of Tim-3 has been reported to boost tumor progression and to be associated with unfavorable prognosis in many types of cancers (Piao et al., 2014; Yan et al., 2015; Zheng et al., 2015; Tawk et al., 2016; Zhang et al., 2019).

Tim-3 has previously been reported to be highly expressed in prostate cancer, hepatocellular carcinoma, and melanoma (Piao et al., 2014; Yan et al., 2015; Zheng et al., 2015; Tawk et al., 2016; Zhang et al., 2019). We initially examined Tim-3 protein levels in GBM tissues using immunohistochemical analysis and observed the expression of Tim-3 in GBM interstitial tissue. In line with previous reports, the present study found that expression of Tim-3 was significantly stronger in GBM samples without MGMT methylation. Tim-3 can efficiently predict the aggressive behavior of head and neck squamous cell carcinomas (Chakravarthi et al., 2014). In prostate cancer, Tim-3 overexpression results in an attenuated level of tumor suppressor FLRT3 and increased expression of genes that trigger invasion and metastasis, such as MMPs (Kim et al., 2017). One study reported that Tim-3 promotes glioma cell proliferation, and increased levels of Tim-3 enhance angiogenesis by inducing transdifferentiation of glioma stem cells into endothelial cells and by stabilizing vascular base membranes, which was implicated as a mechanism by which Tim-3 furthers the progression of gliomas (Hegi et al., 2005). However, that study did not explore the association between Tim-3 levels and prognosis in glioma patients, probably due to the limited number of glioma specimens available. Therefore, the prognostic significance of Tim-3 in glioma remains unclear.

Molecular genetic testing, in particular testing for MGMT promoter methylation, is currently performed to predict the success of standard chemotherapy in GBM (Wick et al., 2010). In one study, 57.14% of responders exhibited MGMT promoter methylation. The same study concluded that patients with MGMT promoter methylation had better outcomes following treatment with temozolomide (Malmstrom et al., 2010). Another single-center study reported that MGMT promoter methylation is an independent prognostic factor for positive outcomes in GBM, including prolonged progression-free survival (PFS) and OS (Dunn et al., 2009). To further investigate the relationship between MGMT promoter methylation status and the expression of Tim-3 in GBM, we retrieved 84 specimens from a tumor tissue bank. In line with previous findings, Tim-3 was expressed at differing levels in GBM tissues. High Tim-3 expression was more frequently observed in GBMs from patients who did not show MGMT promoter methylation, which itself was capable of predicting poor prognosis. Among the 84 patients included in this study, the median OS was 17.3 months. The survival time for the subset of GBM patients with low Tim-3 expression and MGMT promoter methylation was longer than those with moderate or strong Tim-3 expression. Subsequent multivariate cox regression analyses confirmed that Tim-3 expression with MGMT promoter methylation was an independent prognostic factor for GBM patients. The subgroup analysis revealed that strong Tim-3 expression together with MGMT promoter nonmethylation was strongly correlated with shorter survival time. The average survival time, however, hardly differed between patients with unexpressed and those with moderate Tim-3 expression.

We also observed that high-risk GBM patients had higher levels of Tim-3 and unmethylated MGMT. We then constructed an MGMT-associated immune prognostic signature which demonstrated the potential to provide novel insights into the GBM immune microenvironment and possible immunotherapies. This enabled us to classify patients into subgroups with distinct outcomes and immunophenotypes, signifying that this signature may be used to delimit the current prognostic model and facilitate further stratification of patients with GBM and improve the accuracy of prognoses. The results of the current study integrate the complementary values of molecular pathology and immune checkpoint molecule Tim-3 expression to develop a novel model which provides superior survival prediction.

## Conclusion

Our data demonstrate that Tim-3 expression together with MGMT promoter methylation status is correlated with survival in GBM, indicating that Tim-3 is a promising target. Our study assessed the association between clinical prognosis and Tim-3 expression in combination with MGMT promoter methylation. For the first time, we report an association between high levels of Tim-3 expression without MGMT promoter methylation in GBM tissues and worse prognoses. More importantly, univariate and multivariate analyses revealed that a high expression of Tim-3 with MGMT promoter methylation status was a clear prognostic factor for patients with GBM. Moreover, the checkpoint molecule Tim-3 is clearly associated with treatment response and offers prompt, meaningful information for selecting chemotherapeutic drugs.

## Limitations

The current study has several limitations. First, it was a retrospective study. Second, the number of patients was limited. Third, selection bias could not be avoided completely. In addition, there may be other parameters we did not consider that could have influenced the study’s results.

## Data Availability Statement

The raw data supporting the conclusions of this article will be made available by the authors, without undue reservation.

## Ethics Statement

The studies involving human participants were reviewed and approved by the Institutional Review Board of the First Affiliated Hospital of Guangzhou University of Traditional Chinese Medicine. The patients/participants provided their written informed consent to participate in this study.

## Author Contributions

All authors contributed equally to the paper. JZ, KS, XW, and SY drafted the article. LL and YZ performed data collection and statistics. ZC, W-MH, and JL supervised the data collection and revised this article. All authors contributed to the article and approved the submitted version.

## Funding

The study was supported by Project of Guangdong Medical Science and Technology Research Foundation (A2018017), Special fund for clinical research of Wu jieping medical foundation (320.6750.19093-12), Fundamental Research Funds for the Central Universities (19ykpy190), Foundation of the Science and Technology Program of Guangzhou, People’s Republic of China (201607010365), and High Level University Construction Project of Guangzhou University of Traditional Chinese Medicine (A1-AFD018171Z11072). This article has been released as a pre-print at (https://www.researchsquare.com/article/rs-22637/v1).

## Conflict of Interest

The authors declare that the research was conducted in the absence of any commercial or financial relationships that could be construed as a potential conflict of interest.

## Abbreviations

Tim-3, T cell immunoglobulin mucin-3; GBM, glioblastoma multiforme; OS, overall survival; IHC, immunohistochemistry.
